# HIV and AIDS prevention: knowledge, attitudes, practices and health literacy of older persons in the Western Cape and KwaZulu-Natal Provinces, South Africa and in Lesotho

**DOI:** 10.1186/s12877-023-04009-7

**Published:** 2023-05-08

**Authors:** Sebastiana Zimba Kalula, Tarryn Blouws, Maseabata Ramathebane, Abdul-Rauf Sayed

**Affiliations:** 1 The Albertina and Walter Sisulu Institute of Ageing in Africa, University of Cape Town, Cape Town, South Africa; 2 Geriatric Medicine, Department of Medicine, Groote Schuur Hospital, University of Cape Town, Cape Town, South Africa; 3 Pharmacy Department, Faculty of Health Sciences, National University of Lesotho, Maseru, Lesotho; 4 Bristol-Myers Squibb Foundation (BMSF) Technical Assistance Programme, Johannesburg, South Africa

**Keywords:** HIV/AIDs, Knowledge attitudes and practices, Health literacy, Older persons, South Africa, Lesotho

## Abstract

**Background:**

Population ageing and access to anti-retroviral therapies in South Africa have resulted in ageing of the HIV/AIDS epidemic, which has implications for policy, planning and practice. Impactful interventions on HIV/AIDS for older persons require knowledge on effects of the pandemic on this population. A study was undertaken to assess knowledge, attitudes, and practices (KAP) of HIV/AIDS, as well as health literacy (HL) level of a population aged ≥ 50 years.

**Methods:**

A cross-sectional survey was conducted at three sites in South Africa and two sites in Lesotho with an educational intervention at the South African sites. At baseline, data were collected for assessment of KAP of HIV/AIDS and HL levels. The pre- and post-intervention comprised participants at South African sites being familiarised with the contents of a specially constructed HIV/AIDS educational booklet. Participants’ KAP was reassessed six weeks later. A composite score of ≥ 75% was considered adequate KAP and an adequate HL level.

**Results:**

The baseline survey comprised 1163 participants. The median age was 63 years (range 50–98 years); 70% were female, and 69% had ≤ 8 years’ education. HL was inadequate in 56% and the KAP score was inadequate in 64%. A high KAP score was associated with female gender (AOR = 1.6, 95% CI = 1.2–2.1), age < 65 years (AOR = 1.9, 95% CI = 1.5–2.5) and education level (Primary school: AOR = 2.2; 95% CI = 1.4–3.4); (High school: AOR = 4.4; 95% CI = 2.7–7.0); (University/college: AOR = 9.6; 95% CI = 4.7–19.7). HL was positively associated with education but no association with age or gender. The educational intervention comprised 614 (69%) participants. KAP scores increased post intervention: 65.2% of participants had adequate knowledge, versus 36% pre-intervention. Overall, younger age, being female and higher education level were associated with having adequate knowledge about HIV/AIDS, both pre- and post-intervention.

**Conclusions:**

The study population had low HL, and KAP scores regarding HIV/AIDS were poor but improved following an educational intervention. A tailored educational programme can place older people centrally in the fight against the epidemic, even in the presence of low HL. Policy and educational programmes are indicated to meet the information needs of older persons, which are commensurate with the low HL level of a large section of that population.

## Introduction

Population ageing and increased access to anti-retroviral therapies in South Africa are resulting in ageing of the regional HIV and AIDS epidemic. The ageing of the epidemic has implications for policy, planning and practice. Impactful interventions on HIV/AIDS for older persons require knowledge on effects of the pandemic on this population. While the number of studies on prevention of HIV in older persons has increased in high-income countries, few studies have examined how HIV infection may be prevented in this age group in low-income countries [1]. Knowledge is needed to develop and test evidence-based, targeted HIV prevention intervention, and to inform policies and best practices on appropriate prevention programmes for older persons.

While a concerted effort was made in the earlier decades of the HIV epidemic, in the late 1990s to early 2020s, to promote prevention through improvements in health literacy, the education mainly focused on the young population and adults of reproductive age [2–4]. The focus and effort have diminished in the latter two decades however, resulting in even greater exclusion of the older population from prevention programmes. In South Africa, such a focus is particularly relevant for older persons who as a population segment have the lowest levels of health literacy [5, 6] and HIV-related knowledge [7]. Older persons tend not to view themselves susceptible to contracting the virus, keeping in part with attitudes of public and health professionals [8–10]. Ageist attitudes of health professionals are shaped partially by a myth that older adults do not engage in risky behaviours [11], largely viewing them as sexually inactive and therefore not at risk of infection [12, 13]. Older persons have seldom been targeted in prevention programmes, which tend to emphasize voluntary medical male circumcision (VMMC), HIV counselling and testing for younger persons, test-and-treat programmes that provide antiretroviral therapy (ART) to newly infected individuals and conventional ABC (Abstain, Be faithful, Condomise) programmes [2, 3] A large number of the existing HIV prevention programmes are inappropriate for targeting older persons [1, 14]. Scant knowledge is available moreover to inform the design of interventions to target these persons, as well as to inform policy makers about a need (and urgency) of doing so [15].

Referring to the global HIV and AIDS epidemic, the UNAIDS has urged national leaders worldwide to *“*know your epidemic and know your response” [16]. Until now, little attention has been paid to older persons and the HIV/AIDS epidemic in sub-Saharan Africa (SSA) [7]. In 2013, 4.2 million (13%) of adults living with HIV were aged 50 years and older, 50 per cent of whom lived in sub-Saharan Africa [17]. At end 2020, an estimated 36 million people living with HIV/AIDS (PLWHA) globally were aged ≥ 15 years [7] [18]. In 2021, South Africa had approximately 8.2 million (13.7%) people living with HIV (PLWHIV) [19], an increase from 5.38 million (10.6%) in 2011 [20]. A study on prevalence of HIV in persons ≥ 50 years in rural South Africa showed a rate of 16.5 per cent (16.1% females, 17.7% males) [21]. Globally, Lesotho has one of the highest HIV prevalence rates, only second after eSwatini (formerly Swaziland) [22]. The HIV prevalence in persons aged 15–49 years in Lesotho was 20.9 per cent (women 26.1%, men 15.7%) [23]. No information is available currently on HIV prevalence in those aged 50 years and older in Lesotho [15].

Older persons are vulnerable to infectious diseases including HIV and have a poor outcome as has been shown with their increased mortality in the COVID-19 pandemic. Their vulnerability indicates the importance of health education programmes for this population. Attention should neither be drawn away from the equally devastating effects of the HIV pandemic. A fairly radical shift took place in health care policy, practice and priorities in 2019/2020, towards fighting the COVID-19 pandemic [24]. The diagnosis, treatment and education of HIV/AIDS was eclipsed, or halted somewhat, to focus on preventing and treating COVID-19 [25, 26].

The number of older persons living with HIV in sub-Saharan Africa is expected to increase by more than 200 per cent over the coming three decades [27, 28]. Population ageing in the sub-continent will exacerbate the existing HIV situation, particularly in that little is known of persons who become infected with HIV in later life, or who age with HIV having contracted the virus earlier in life.

Knowledge about HIV transmission and prevention accompanied by a reduction in behavioural risk practices are important in combatting the spread of HIV. Several factors contribute to the rate of new HIV infections: i) Personal factors, such as a lack of knowledge and skills required to protect oneself and others; ii) quality and coverage of service factors, such as inaccessibility due to distance, cost and poor health provider interactions; and iii) societal factors, such as social and cultural norms, practices, beliefs and laws that stigmatize and disempower certain groups, acting as barriers to HIV prevention campaigns [29]. Older persons have unique biological and psychosocial vulnerabilities to HIV infection [30]. Yet little is known about HIV risk and protective factors in this age group, and even less about prevention practices. Nonetheless, a growing consensus is that prevention and improved health care access through health literacy constitute a cornerstone in preventing infection in the age group [31–33].

Health literacy is a key tool in interventions to prevent and combat epidemics such as HIV. The World Health Organisation (WHO) describes health literacy as “the cognitive and social skills which determine the motivation and ability of individuals to gain access to, understand and use information in ways which promote and maintain good health” [34]. As such, it entails people’s knowledge, motivation and competences to access, understand, think through and apply health information in order to make judgments and take decisions in everyday life concerning health care to maintain or improve quality of life across the life course [31]. Low health literacy has been reported to be associated with increased mortality [35], hospitalisation, lower use of preventive healthcare services [36], poor adherence to prescribed medications, difficulty communicating with health professionals, and poorer knowledge about disease processes and self-management skills among people with chronic conditions [37]. Health literacy capacity is mediated by education, and its adequacy is affected by culture, language and the characteristics of health-related settings [38]. Overall literacy skills include ability to access and comprehend print and oral communication mediums, pertaining to both literacy and numeracy. Hence, whereas an individual requires an ability to read, write and comprehend written language [39] to benefit from a prevention programme, an assumption of health literacy, particularly in the older population, in low- to middle-income countries may not be feasible in all cases. Programmes therefore need to be specifically tailored to meet health literacy deficiencies in this population for them to be effective.

Prevention practices are the outcome component of the knowledge, attitudes and practices (KAP) framework within the conceptual model. KAP surveys are widely used in research globally to investigate behavioural and social change processes, and to establish correlates of health behaviour outcomes. *Knowledge* is defined as a set of facts and understanding that helps to identify gaps in information and education. *Attitudes* are the positions people hold which mediate between a situation and a response; they help to explain why people exposed to the same stimulus adopt different practices. *Practices* are the observable actions (behaviours) that occur in response to a stimulus [40].

The literacy rate of the population aged 65 years and over in South Africa was reported as 54.5 per cent in 2017 [41] and that of Lesotho, 60.5 per cent in 2015 [42]. It has been reported moreover that only 22.4 per cent of persons aged 50 + years in the country have accurate knowledge of sexual transmission of HIV and understand the fallacy of misconceptions of HIV transmission [3]. This population’s knowledge in this regard is reported to have declined since 2008–2012 [3] moreover, which supports a need to improve prevention practices. Older people’s health behaviour correlates with their knowledge, attitudes and practices. Given the scant research and evidence of a low level of knowledge on HIV and AIDS in the older population in South Africa and Lesotho, a study was undertaken of the knowledge, attitudes and practices of HIV and individual-level health literacy of older persons in these two countries.

## Methods

### Study sites and study population

The study was designed as a cross-sectional, quantitative survey, followed by an educational intervention and a subsequent post-intervention survey. The project was carried out in two phases: Phase 1—Development of a questionnaire to collect data for the quantitative survey, training of field workers, and collection of quantitative data on HIV and AIDS knowledge, attitudes and practices; and Phase 2 – An educational intervention on HIV and AIDS, and a follow-up assessment of HIV and AIDS knowledge, attitudes and practices. The study was primarily located at sites in South Africa with additional sites in the neighbouring state of Lesotho, an independent state geographically located within South Africa. The pre-post intervention study was conducted at sites in South Africa, but not in Lesotho.

The study was conducted between January 2016 and December 2018. Study sites in South Africa were the Cape Winelands district and Vredenburg in the West Coast district in the Western Cape, and eDumbe, Paulpietersburg in KwaZulu-Natal; and in Lesotho, in the Maseru and Thaba-Tseka districts. Except for the Cape Winelands, the sites were selected based on the presence of non-governmental organisations (NGOs) which provide services to older persons in these communities. The NGOs operate seniors’ centres that offer a venue for older persons to congregate, and to benefit from services and activities. The NGOs enabled access moreover to eligible participants.

In addition, the sites were selected based on the size (percentage) of the older population: Cape Winelands 13%; Vredenburg district, 19%; eDumbe KwaZulu-Natal, 13% [43, 44]. In Lesotho the percentages were: Maseru district 12% and Thaba-Tseka district 14%) [45]. Site selection and co-ordination of the research were planned in collaboration with two non-governmental organisations and a training institution: Ukuzakha Nokuzenzela Women’s Association (UKUNOWO) (KwaZulu-Natal) and Mfesane (Vredenburg, West Coast), and the Department of Pharmacology at the University of Lesotho. The study population was persons aged ≥ 50 years resident at the study sites.

The survey was community based. The sampling method was non-random, convenience sampling due to the relative low proportion of the older population in the study community. Fieldworkers visited households in the study site to recruit participants who met the inclusion criteria. The inclusion criteria were residents in a household aged 50 years and older who were able to give consent to participate in the study. Persons unable to communicate, or to understand the informed consent requirement were excluded from participation. Approval for this study was obtained from the Human Research Ethics Committee of the Faculty of Health Sciences, University of Cape Town (HREC REF 472/2015). Informed Consent was obtained from participants prior to enrolment in the study and the study conduct complied with the Helsinki Declaration of 2013.

### Sample size

We anticipated that the proportion with adequate or comprehensive knowledge of HIV and AIDS prevention would be approximately 22% [3] (*P* = 0.22; d = 0.05 is the desired precision). The sample size was calculated using the formula for estimating a population proportion, *n* = p(1 − p)(1.96)^2^ ÷ d^2^ [46]. The minimum sample size required for this study was 264 at each site, in both South Africa and Lesotho. A total sample size of 1163 participants aged ≥ 50 years was achieved and employed.

The two phases of the study were as follows:

### Phase 1

#### Questionnaire development

No validated questionnaire was available to assess knowledge, attitudes, and practices (KAP) of HIV/AIDS in the local population. A structured questionnaire was constructed for the study which included a validated questionnaire used previously in global studies [47]. The baseline quantitative survey instrument provided for collection of data in the following domains: Demographic characteristics; medical history (self-reported medical conditions as diagnosed by a medical practitioner which were being clinically treated); and questions pertaining to subjects’ knowledge, attitudes and practices in relation to HIV and AIDS. The questionnaire and an accompanying informed consent form were translated into Afrikaans, isiXhosa, isiZulu and Sesotho, languages mainly spoken in the study communities.

##### Knowledge, Attitudes and Practices (KAP)

The KAP items in the questionnaire were adapted from the International Aids Questionnaire-English Version (IAQ-E) which has been used on college students [47]. The IAQ-E is an 18-item questionnaire, of which 16 items were adapted and used in the study, hereafter referred to as the 16-item questionnaire. The two items omitted were: “People with HIV should be kept out of school” and “I would end my friendship if my friend had AIDS.” The items were deemed inappropriate for the older study participants. The IAQ-E provides for assessment of awareness of HIV/AIDS by four factors: 1) Misconceptions about HIV transmission; 2) prejudice/attitudes towards persons infected with the virus; 3) perceived personal risk of infection; and 4) factual knowledge about HIV/AIDS.

##### Health literacy

The health literacy questions in the questionnaire were adapted from the Short Test of Functional Health Literacy in Adults (STOFHLA), a brief screening health literacy questionnaire that consists of 16 questions (score of 0–16). This assessment was selected based on domains that were identified for participants with limited health literacy: Navigating the healthcare system, completing medical forms, following medication instructions, interacting with providers, and reading appointment cards [48]. The questions were phrased to ask participants “how often” they had a problem, or “how confident” they felt in each of the domains, rather than asking “if” they had a problem.

The questionnaire was piloted with five men and five women at each of the five study sites to review content and clarity, obtain feedback, and gauge time required to complete an interview. The instrument was modified accordingly.

### Training

Data collection for the study commenced in January 2016. Field workers were recruited from organisations at the sites. The organisations in South Africa were AandBlom and Charleston Hill Senior Klub (a seniors’ clubs) in the Cape Winelands; Mfesane (a non-profit organisation) in Vredenburg on the West Coast; and Ukuzakha Nokuzenzela Women’s Association (UKUNOWO) in KwaZulu-Natal. The University of Lesotho already had trained field workers who were recruited for the study.

Both the project leader and the researcher travelled to each site (except the Lesotho sites) for training of the field workers. The training covered: Understanding the purpose and context of the study; expectations held of field workers in acquitting their task; understanding the questionnaire and its contents; and problem solving and conflict resolution during recruiting and interviewing participants. Mock interviews were conducted by the field workers between one another, for practice and as part of the training. The number of field workers trained were: Four females in the Cape Winelands, eight females in KwaZulu-Natal; and nine females and one male in Vredenburg, three females and three males in Lesotho.

### Data collection

#### Baseline study

Collection of the data was overseen by the researcher in the Western Cape, and by a study co-ordinator at the KwaZulu-Natal and Lesotho sites. The researcher collected completed questionnaires from field workers at the Cape Winelands and Vredenburg sites and conducted quality control checks on the completed questionnaires. The KwaZulu-Natal and Lesotho sites’ questionnaires were sent to the researcher by post. The data from each site were entered into separate Microsoft Access databases.

### Phase 2

#### Intervention (follow-up) study

A follow-up study was conducted among study participants after completion of the survey and analysis of the data collected in phase 1. The follow-up study took the form of an intervention, a pre-post design, to assess the impact of basic HIV/AIDS education on the KAP score of the study participants.

Following on an analysis of the quantitative data collected during phase 1, an information booklet with pertinent information on HIV and AIDS to be imparted to all participants was compiled in English and translated into Afrikaans, isiXhosa and isiZulu.

Field workers at all sites at which phase 1 of the study was conducted, excluding field workers in Lesotho, were re-trained in the purpose and objectives of the follow-up intervention study. The field workers in the Cape Winelands [3], in KwaZulu-Natal [8] and in Vredenburg [9] were trained in the contents of the booklet, and how the booklet and the 16-item questionnaire were to be administered to the study participants. In the intervention, field workers instructed participants face-to-face on the information in the booklet, in a participant’s preferred language, and left a booklet with each participant for future reference. Participants were given the opportunity to ask questions during the session with the field worker. The participants typically reside in multi-generational households, and if illiterate or visually impaired, were able to ask a family member to familiarise them with the contents.

A follow-up questionnaire was constructed and administered to the participants during a visit approximately six weeks after the participants had been introduced to the booklet. Essentially, the follow-up questionnaire focused on knowledge, attitudes and practices, including perceptions, of HIV and AIDS in general (the 16-item questionnaire) [47]. No consultation of the booklet was permitted at the time of the interview. Thereafter, a comparison was made between the KAP data collected pre-intervention and the data collected post-intervention (i.e., after distribution of the information booklet) to determine any change in gaps in knowledge, awareness, attitudes, and behavioural practices.

### Statistical analyses

Data were captured in an excel spread sheet and exported to Stata 13 [49] for analysis. Numerical variables are expressed as median and range, and categorical variables as frequency tables. McNemar's test was used to compare pre- and post-intervention data. An association between level of knowledge and demographic characteristics (gender, age and education) was assessed using bivariate and multivariate logistic regression analysis. Odds ratios (OR) and 95% confidence intervals (CI) were used to measure association for bivariate analysis and adjusted odds ratios (AOR) were used for multivariate analysis. For all analyses, a *p*-value of less than 0.05 and a 95% confidence interval that does not span unity were considered as thresholds of statistical significance.

Responses to the 16-item questionnaire that measured four areas of HIV/AIDS awareness – factual knowledge, misconceptions, prejudice and personal risk—were coded 1 for a correct response, and 0 for an incorrect or a “Don’t know” response. A composite KAP score ≥ 75% was considered “Adequate knowledge”.

## Results

### Demographic characteristics

A total of 1163 questionnaires were completed at the four sites in phase 1 of the study: Cape Winelands (*n* = 297, 25.5%), Vredenburg (*n* = 300, 25.8%), KwaZulu-Natal (*n* = 298, 25.6%) and Lesotho (*n* = 268, 23%). Thirty per cent of the study participants were male (*n* = 349) and 70 per cent were female (*n* = 814). The median age of the participants was 63 years (range 50–98 years).

The youngest study subsample (median age 56 years; range 50–76) was drawn in Vredenburg, on the West Coast. Sixty-eight per cent of the participants here were in the age group 50–59 years; 25 per cent were still employed. Paarl in the Cape Winelands had the highest percentage of older participants with a median age of 68 years (range 53–91 years). Twelve per cent of the Paarl (Cape Winelands) samples were aged 80 years and over (Table 1).

**Table 1:** Demographic characteristics of participants by four study sites (*n* = 1163)

	**Cape Winelands ** ***N*** ** = 297**		**KwaZulu-Natal ** ***N*** ** = 298**		**Lesotho ** ***N*** ** = 268**		**Vredenburg ** ***N*** ** = 300**		**Total ** ***N*** ** = 1163**	
***Median age (range) years***	68	(53—91)	62	(50—98)	67	(50 – 92)	56	(50 – 76)	63	(50 – 98)
	***N***	***(%)***	***N***	***(%)***	***N***	***(%)***	***N***	***(%)***	***N***	***(%)***
***Age group (years)***										
50 – 59	34	(11.5)	118	(39.6)	75	(28.0)	204	(68.0)	431	(37.1)
60 – 69	132	(44.4)	98	(33.0)	86	(32.1)	79	(26.3)	395	(34.0)
70 – 79	95	(31.9)	57	(19.1)	84	(31.3)	17	(5.7)	253	(21.7)
80^+^	36	(12.2)	25	(8.4)	23	(8.6)	0	(0)	84	(7.2)
***Sex***										
Male	81	(27.3)	61	(20.5)	81	(30.2)	129	(43)	352	(30.3)
Female	216	(72.7)	237	(79.5)	187	(69.8)	171	(57)	811	(69.7)
***Marital status***										
Married	123	(41.1)	0	(0.0)	86	(32.1)	134	(44.6)	343	(29.5)
Widow	114	(38.4)	87	(29.2)	164	(61.2)	73	(24.2)	438	(37.7)
Single	33	(11.1)	114	(38.3)	9	(3.4)	67	(22.3)	223	(19.2)
Divorced	12	(4.0)	7	(2.4)	8	(3.0)	8	(2.7)	35	(3.0)
Cohabiting	15	(5.1)	90	(30.2)	1	(0.4)	18	(6.0)	124	(10.6)
***Education***										
No schooling	41	(13.8)	46	(15.4)	28	(10.5)	57	(19.0)	172	(14.8)
Primary school	139	(46.8)	145	(48.7)	188	(70.2)	158	(52.7)	630	(54.2)
High school	98	(33.0)	83	(27.9)	46	(17.2)	81	(27.0)	308	(26.5)
University/College	19	(6.4)	24	(8.1)	6	(2.2)	4	(1.3)	53	(4.5)
***Employment***										
Yes	21	(7.1)	41	(13.8)	18	(6.7)	76	(25.3)	156	(13.4)
No	276	(92.9)	257	(86.2)	250	(93.3)	224	(74.7)	1007	(86.6)
***Social grant (pension)***										
Yes	247	(83.2)	166	(55.7)	104	(38.8)	120	(40)	637	(54.8)
No	45	(15.1)	130	(43.6)	164	(61.2)	179	(59.7)	518	(44.5)
Unknown	5	(1.7)	2	(0.7)	0	0	1	(0.3)	8	(0.7)

#### Education level

Slightly over half (54%) of the total sample had a primary school education and 15 per cent had no schooling; 5 per cent had a tertiary education. The highest percentage (19%) of participants with no schooling was sampled in Vredenburg, while the highest percentage with a tertiary education (8.1%) was sampled in KwaZulu-Natal.

#### Employment

Rates of employment were not expected to be high as the study population comprised largely retired persons or persons no longer economically active. The highest percentage of those still in employment was drawn in Vredenburg (25%), while the highest percentage not in employment (93%) was drawn in Lesotho. Over half of the total sample (55%) received a means tested social older person's grant (in 2020, the amount of the grant payable monthly to eligible persons aged ≥ 60 years in South Africa was R1 860 (US$111); in Lesotho, in the same year, the amount payable monthly was M750 (US$45), but only to beneficiaries aged ≥ 70 years).

### Medical/Chronic conditions

**Fig. 1:**
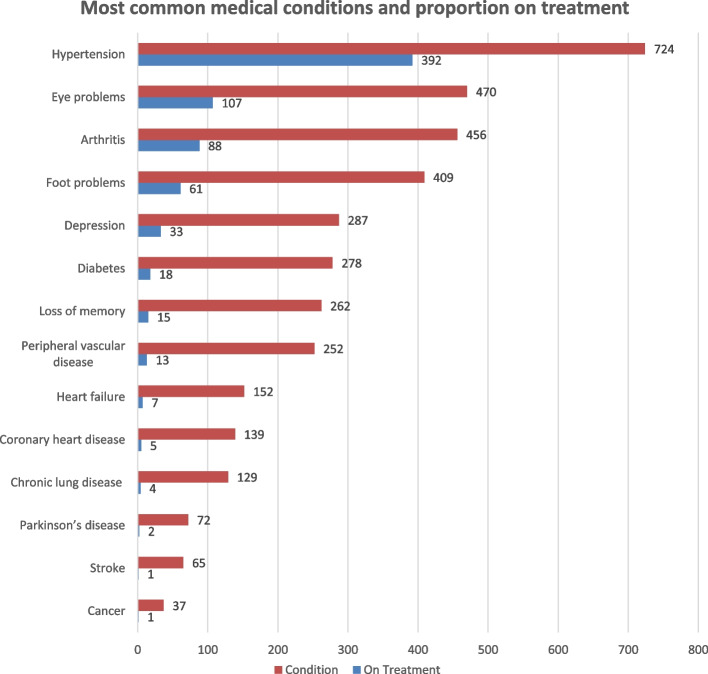
Numbers of participants with self-reported medical conditions and proportions of the conditions being clinically treated

The most common self-reported medical conditions, reported as being diagnosed by a medical practitioner, were hypertension (*n* = 724, 62%), eye problems (*n* = 470, 40%), arthritis (*n* = 456, 39%), foot problems (*n* = 409, 35%), depression (*n* = 287, 25%), diabetes mellitus (*n* = 278, 24%) and memory loss (*n* = 262, 22.5%) (Fig. 1). Only small percentages of participants who reported these conditions were receiving treatment for the condition: hypertension (54%), arthritis (23%), diabetes (19%), eye problems (15%), foot problems (12%), depression (5%) and memory loss (3%) (Fig. 1).

### Health literacy

Health literacy (HL) items used in the survey were scored on a Likert scale 1–5: a score of 1 indicating “Always”; of 2, “Often”; of 3, “Sometimes”; of 4, “Occasionally;” and of 5, “Never”. Some items required reverse scoring so that each of the 16-item health literacy questions were consistent when calculating an overall mean score. Thirty-seven participants (3.2%) had incomplete HL data. The Shapiro–Wilk test indicated that the distribution of the HL scores was not normally distributed; hence, a binary variable was created (a score ≥ 3.75 out of 5 (75%) was considered as 'Adequate HL level' and < 3.75 as ‘Inadequate HL level') [50]. Overall, health literacy was inadequate in 56 per cent of the sample (*n* = 1126) and adequate in 44 per cent. The lowest literacy levels were recorded in the Vredenburg participants, with inadequate HL at 73 per cent. Vredenburg, it must be noted, had the highest percentage of participants with no schooling (19%). The highest percentages of those with adequate health literacy level were reported in KwaZulu-Natal and the Cape Winelands, at 60 per cent and 55 per cent, respectively.

### HIV knowledge, attitudes, and practices

Overall, HIV/AIDS awareness (KAP score) was inadequate (score < 75%) in approximately two-thirds (64%) of the total sample. The Cape Winelands (74%) and the Lesotho (69%) sites had the highest percentages of participants with inadequate KAP scores. Participants at the KwaZulu-Natal and Vredenburg sites had the highest percentages with adequate knowledge: 45% and 41%, respectively.

Multiple logistic regression analysis found a significant association between the outcome variable, KAP score (adequate knowledge of HIV/AIDS, yes/no) and demographic variables (age, gender, and education). Being female and being a younger older adult (< 65 years) were almost twice as likely to have a high KAP score (AOR = 1.6; 95% CI: 1.2–21) and (AOR = 1.9; 95% CI: 1.5–2.5). For education, the odds of a high KAP score increased with higher level of education (Primary school: AOR = 2.2; 95% CI: 1.4–3.4); (High school: AOR = 4.4; 95% CI: 2.7–7.0); (University /college: AOR = 9.6; 95% CI: 4.7–19.7).

For health literacy, there was a significant association with education, but not with gender and age. Adequate knowledge of health literacy (yes/no) was associated with higher level of education. The odds of adequate knowledge of health literacy increased with higher level of education (Primary school**:** AOR = 4.0; 95% CI: 2.5–6.4); High school: AOR = 9.9; 95% CI: 6.1–16.2); (University /college: AOR = 24.3; 95% CI: 10.7–55.0).

### The intervention

#### Post-intervention participation

All research sites, excluding the Lesotho sites, were included in the intervention study. The Lesotho sites were excluded for resource and logistical reasons. With the exclusion of the Lesotho sample, 895 of the remaining phase 1 study participants were eligible for inclusion in the intervention study. However, only 614 (69%) participants were successfully recruited for the follow-up study: Of the subsamples, Cape Winelands 44% (130/297), KwaZulu-Natal 80% (239/298) and Vredenburg 82% (245/300). Reasons for a significant decline in participation in the follow-up study included: i) Death, relocation and refusal to participate for a second time (these reasons were most commonly given at all sites); and ii) an outbreak of bird flu in the Cape Winelands and on farms at the time of the follow-up study, which prevented the researcher and field workers from visiting the farms. There was no major difference in the demographic characteristic between participants and drop-outs, except for drop-outs being older, widowed, and likely to receive a means tested social grant (Table 2). (Beneficiaries of a social grant must be aged 60 years or older).

**Table 2:** Demographic characteristics of study participants and those lost to follow-up (drop-outs)

Demographic characteristics	Post-intervention participation		Participants lost to follow-up (dropouts)		*P*-value
***Median age (range) years***	***n*** ** = 614**		***n*** ** = 281**		
Median	60		65		< 0.001
Range	50–96		50–98		
**Age-group**	**N**	**%**	**N**	**%**	
≤ 60	313	51	89	31.7	< 0.001
> 60	301	49	192	68.3	
**Sex**					
Male	185	30.1	86	30.6	0.886
Female	429	69.9	195	69.4	
**Marital status**					
Married	172	28.0	85	30.3	< 0.001
Widowed	162	26.4	110	39.2	
Single	168	27.4	49	17.4	
Divorced	16	2.6	11	3.9	
Cohabiting	96	15.6	26	9.3	
**Education level**					
Primary/no school	410	66.8	176	62.6	0.226
High school/tertiary	204	33.2	105	37.4	
**Social grant (pension)**					
Yes	331	53.9	202	71.9	< 0.001
No	276	45.0	78	27.8	
Unknown	7	1.1	1	0.4	

### Post-intervention data analysis and results

Results of an analysis of the post-intervention data are presented as a comparison of pre- and post-intervention phase outcomes on the KAP scores obtained from the participating sites.

### Overall KAP score

Responses to the 16 items on Knowledge, Attitude and Practices, a composite score > 8 is regarded as above average knowledge. The percentages scoring above average between the pre- and the post-intervention studies were compared and are shown in Table 3 by site and overall difference in the average scores.

**Table 3:** Comparison of KAP scores (pre- and post-intervention): percentages with an above average score

Above average KAP knowledge	Cape Winelands (*n* = 130)	KwaZulu-Natal (*n* = 239)	Vredenburg (*n* = 245)	Total (*n* = 614)
Pre-intervention	45.4	81.6	71.8	70.0
Post-intervention	44.6	97.1	82.9	80.3
**Difference**	**-0.8**	**15.5**	**11.0**	**10.3**
*P-value*	*0.889 (NS)*	< *0.001*	*0.001*	< *0.001*

*P*-value (McNemar's test), *NS* Not statistically significant

The results show a significant (10%) overall improvement in the KAP score, particularly at two sites: KwaZulu-Natal with an almost 16 per cent improvement and Vredenburg with an 11 per cent improvement. The Cape Winelands’ results show a non-significant decline in the KAP score.

### Post-intervention KAP scores

Using the composite score (≥ 75%) for knowledge, almost two thirds (65.2%) of the participants had adequate knowledge of HIV and AIDS, and prevention from contracting the virus, compared to 36 per cent pre-intervention. The percentage with adequate knowledge in KwaZulu-Natal increased from 45 to 90 per cent, and in Vredenburg, from 41 to 64 per cent. The degree of improvement in responses to individual items ranging from highest to lowest percentages of improvement in the pre-and post-intervention responses is shown in Table 4

**Table 4:** Correct responses to knowledge, attitudes, and practices (KAP), pre- versus post- intervention (percentages and differences), in descending order of difference

Item type		Correct answer (%)		
		Pre-	Post-	Diff
Myth	Mosquitoes can transmit HIV	32.1	69.1	37.0
Personal risk	Older persons are less susceptible to contracting HIV than persons in other age groups	41.7	70.4	28.7
Personal risk	You can protect yourself against HIV/AIDS by being vaccinated against it	48.4	64.8	16.4
Myth	HIV/AIDS can be spread through coughing and sneezing	66.1	81.6	15.5
Myth	HIV/AIDS can be contracted from toilet seats	65.3	79.2	13.9
Myth	HIV/AIDS can be contracted through sharing cigarettes	67.3	80.6	13.3
Personal risk	HIV/AIDS only affects drug users, homosexuals, and prostitutes	56.8	67.4	10.6
Myth	HIV/AIDS can be transmitted through the air	74.4	84.9	10.5
Attitude	If a family member contracts HIV, he/she should move out of the home	70.2	80.5	10.3
Myth	HIV/AIDS can be spread through hugging an infected person	79.2	87.5	8.3
Attitude	People with HIV/AIDS should stay home or in a hospital	30.3	38.3	8.0
Myth	HIV/AIDS can be spread at swimming pools	71.0	78.5	7.5
Fact	HIV/AIDS can be transmitted from mother to baby	71.7	77.0	5.3
Fact	HIV/AIDS is spread through infected sperm	84.0	88.3	4.3
Fact	Condoms decrease the risk of HIV transmission	78.8	80.0	1.2
Attitude	I am willing to do volunteer work with HIV/AIDS patients	53.9	54.6	0.7

*Diff * magnitude of change in proportion to correct answers

KAP scores pre- versus post- intervention by study site showed the Cape winelands to score lower than other sites on all items except the item ‘HIV/AIDS is spread through infected sperm’ (Table 5).

**Table 5:** Correct responses regarding knowledge, attitudes and practices (pre- versus post- percentages)

KAP Questions	Cape Winelands (*n* = 130)	KwaZulu-Natal (*n* = 239)	Vredenburg (*n* = 245)	Total (*n* = 614)
HIV/AIDS can be spread through coughing and sneezing				
Pre-intervention	46.2	71.1	71.8	66.1
Post-intervention	50.8	92.1	87.8	81.6
**Difference**	**4.6**	**20.9**	**15.9**	**15.5**
*P-value*	*0.4227 (NS)*	< *0.001*	< *0.001*	< *0.001*
HIV/AIDS can be contracted through sharing cigarettes				
Pre-intervention	41.5	71.6	76.7	67.3
Post-intervention	47.7	95.4	83.7	80.6
**Difference**	**6.2**	**23.9**	**6.9**	**13.4**
*P-value*	*0.285 (NS)*	< *0.001*	*0.0269*	< *0.001*
HIV/AIDS can be spread through hugging an infected person				
Pre-intervention	61.5	87.0	80.8	79.2
Post-intervention	62.3	96.2	92.2	87.5
**Difference**	**0.8**	**9.2**	**11.4**	**8.3**
*P-value*	*0.884 (NS)*	< *0.001*	< *0.001*	< *0.001*
HIV/AIDS can be transmitted through the air				
Pre-intervention	50.8	82.4	79.2	74.4
Post-intervention	54.6	97.9	88.2	84.9
**Difference**	**3.8**	**15.5**	**9.0**	**10.4**
*P-value*	*0.4922 (NS)*	< *0.001*	*0.0052*	< *0.001*
HIV/AIDS can be spread through swimming pools				
Pre-intervention	46.2	79.5	75.9	71.0
Post-intervention	44.6	95.8	79.6	78.5
**Difference**	**-1.5**	**16.3**	**3.7**	**7.5**
*P-value*	*0.7963 (NS)*	< *0.001*	*0.3051 (NS)*	*0.001*
HIV/AIDS can be contracted through toilet seats				
Pre-intervention	42.3	72.8	70.2	65.3
Post-intervention	42.3	97.1	81.2	79.2
**Difference**	**0.0**	**24.3**	**11.0**	**13.8**
*P-value*	*0.999 (NS)*	< *0.001*	*0.0027*	< *0.001*
Mosquitos can transmit HIV				
Pre-intervention	26.9	28.5	38.4	32.1
Post-intervention	27.7	89.5	71.0	69.1
**Difference**	**0.8**	**61.1**	**32.7**	**37.0**
*P-value*	*0.884 (NS)*	< *0.001*	< *0.001*	< *0.001*
I am willing to do volunteer work with HIV/AIDS patients				
Pre-intervention	46.9	65.7	46.1	53.9
Post-intervention	36.2	69.9	49.4	54.6
**Difference**	**-10.8**	**4.2**	**3.3**	**0.7**
*P-value*	*0.0477 (NS)*	*0.2113 (NS)*	*0.4371*	*0.7874 (NS)*
If a family member contracts HIV, he/she should move out				
Pre-intervention	38.5	86.6	71.0	70.2
Post-intervention	55.4	93.7	80.8	80.5
**Difference**	**16.9**	**7.1**	**9.8**	**10.3**
*P-value*	*0.002*	*0.0065*	*0.0114*	< *0.001*
People with HIV/AIDS should stay home or in a hospital				
Pre-intervention	36.2	13.4	43.7	30.3
Post-intervention	29.2	16.3	64.5	38.3
**Difference**	**-6.9**	**2.9**	**20.8**	**8.0**
*P-value*	*0.2249 (NS)*	*0.327 (NS)*	< *0.001*	*0.001*
Older people are less susceptible of contracting HIV than other age groups				
Pre-intervention	36.9	31.0	54.7	41.7
Post-intervention	32.3	83.3	78.0	70.4
**Difference**	**-4.6**	**52.3**	**23.3**	**28.7**
*P-value*	*0.4227 (NS)*	< *0.001*	< *0.001*	< *0.001*
HIV/AIDS only affects drug users, homosexuals and prostitutes				
Pre-intervention	47.7	59.8	58.8	56.8
Post-intervention	53.9	77.0	65.3	67.4
**Difference**	**6.2**	**17.2**	**6.5**	**10.6**
*P-value*	*0.285 (NS)*	< *0.001*	*0.0736*	< *0.001*
You can protect yourself against HIV/AIDS by being vaccinated for it				
Pre-intervention	26.9	69.5	39.2	48.4
Post-intervention	33.1	89.5	57.6	64.8
**Difference**	**6.2**	**20.1**	**18.4**	**16.5**
*P-value*	*0.2673 (NS)*	< *0.001*	< *0.001*	< *0.001*
Condoms will decrease the risk of HIV transmission				
Pre-intervention	70.0	90.0	72.7	78.8
Post-intervention	63.9	98.7	70.2	80.0
**Difference**	**-6.2**	**8.8**	**-2.5**	**1.1**
*P-value*	*0.2278 (NS)*	< *0.001*	*0.5176 (NS)*	*0.5739 (NS)*
HIV/AIDS can be transmitted from mother to baby				
Pre-intervention	75.4	82.4	59.2	71.7
Post-intervention	70.8	84.5	73.1	77.0
**Difference**	**-4.6**	**2.1**	**13.9**	**5.4**
*P-value*	*0.3657 (NS)*	*0.5472 (NS)*	< *0.001*	*0.018*
HIV/AIDS is spread through infected sperm				
Pre-intervention	88.5	96.2	69.8	84.0
Post-intervention	86.2	98.3	79.6	88.3
**Difference**	**-2.3**	**2.1**	**9.8**	**4.2**
*P-value*	*0.5637 (NS)*	*0.1655 (NS)*	*0.0088*	*0.020*

*P*-value (McNemar's test)

*NS *Not statistically significant

### Association between post-intervention KAP and demographic characteristics

A significant association between the outcome variable (adequate knowledge of HIV and AIDS prevention (yes/no)) and selected demographic variables (age, gender and education) was observed in the bivariate and multivariate analysis. When analysed by region, the selected demographic characteristics were significant predictors of knowledge among participants in Vredenburg. Among participants in KwaZulu-Natal, only education was a significant predictor of knowledge when adjusted for all demographic variables. For the Cape Winelands, an association was found with age on bivariate analysis, but no association on multivariate analysis (Table 6).

**Table 6:** Association between post-intervention knowledge and demographic characteristics of the sample

**Demographic characteristic**	**Adequate HIV/ AIDS prevention knowledge**				
**All regions combined (** ***n*** ** = 614)**	**No (%)**	**Yes (%)**	**OR (95% CI)**	**AOR (95%CI)**	***P*** **-value**
**Age-group**					
> 60 years	137 (45.5)	164 (54.5)			
≤ 60 years	77 (24.6)	236 (75.4)	2.5 (1.8—3.6)*	2.6 (1.9–3.7)*	< 0.001
**Gender**					
Male	75 (40.5)	110 (59.5)			
Female	139 (32.4)	290 (67.6)	1.4 (1.0—2.0)*	1.6 (1.1–2.4)*	0.013
**Education level**					
Primary/no schooling	155 (37.9)	254 (62.1)			0.043
High school/tertiary	59 (28.8)	146 (71.2)	1.5 (1.1—2.2)*	1.5 (1.0–2.1)*	
**Cape Winelands (** ***n*** ** = 130)**					
**Age-group**					
> 60	92 (80.7)	22 (19.3)			
≤ 60	9 (56.2)	7 (43.8)	3.2 (1.1- 9.4)*	3.0 (0.9–9.4)	0.058
**Gender**					
Male	23 (69.7)	10 (30.3)			
Female	78 (80.4)	19 (19.6)	0.6 (0.2—1.3)	0.5 (0.2–1.4)	0.194
**Education**					
Primary and no school	63 (82.9)	13 (17.1)			
High school and above	38 (70.4)	16 (29.6)	2.0 (0.9—4.6)	1.6 (0.7–3.9)	0.243
**KwaZulu-Natal (** ***n*** ** = 239)**					
**Age-group**					
> 60	16 (12.8)	109 (87.2)			
≤ 60	9 (7.9)	105 (92.1)	1.7 (0.7—3.9)	1.5 (0.6–3.6)	0.336
**Gender**					
Male	7 (14.3)	42 (85.7)			
Female	18 (9.5)	172 (90.5)	1.6 (0.6—3.9)	1.7 (0.7–4.5)	0.273
**Education**					
Primary and no school	21 (13.6)	133 (86.4)			
High school and above	4 (4.7)	81 (95.3)	3.2 (1.1—9.2)*	3.1 (1.0–9.4)*	0.047
**Vredenburg (** ***n*** ** = 245)**					
**Age-group**					
> 60	29 (46.8)	33 (53.2)			
≤ 60	59 (32.2)	124 (67.8)	1.8 (1.0—3.3)*	1.9 (1.1–3.5)*	0.033
**Gender**					
Male	45 (43.7)	58 (56.3)			
Female	43 (30.3)	99 (69.7)	1.8 (1.1—3.0)*	1.8 (1.1–3.2)*	0.028
**Education**					
Primary and no school	71 (39.7)	108 (60.3)			
High school and above	17 (25.8)	49 (74.2)	1.9 (1.0—3.5)*	1.8 (0.9–3.4)	0.065
*OR: Odds ratio*					

*AOR *Adjusted odds ratio

*95% CI *95% Confidence interval

## Discussion

The study set out to investigate the knowledge, attitudes and practices of a sample of older persons regarding HIV/AIDS, and the sample’s level of health literacy in general. Information on self -reported health conditions as diagnosed by a health practitioner and subsequent clinical treatment of the condition/s was collected to gauge participants’ interaction with the health care system. The participants reported a number of chronic non-communicable diseases, the majority of which were not being treated clinically. Both knowledge of HIV/AIDS scores and health literacy scores were low**.** It should be noted that seventy per cent of the sample had only a primary school level of education, and 15 per cent had no schooling**.**

Multi-morbidity, poor chronic disease control and unmet treatment needs in older populations, particularly in developing regions, have been reported widely [51–54]. Broadly, the studies have found that awareness, treatment, and control of chronic diseases are lower in populations with low literacy and low socio-economic status, which findings are consistent with those of our study.

The level of knowledge of HIV/AIDS in our study was positively associated with the sample’s level of education and negatively associated with age (the median age of the sample was 63 years (range 50–98 years)). In our study, the Cape Winelands participants had the lowest KAP score, but the highest percentage of older participants, with a median age of 68 years (range 53–91 years). Twelve per cent of the Paarl (Cape Winelands) samples were aged 80 years and over.

Except for the Cape Winelands, the KAP score improved after an appropriately designed education intervention which addressed myths, facts and risk factors for HIV/AIDS. The low scores in the Cape Winelands are likely a function of a large number lost to follow-up and lack of access of participants to a formal senior centre where members are exposed to some educational activities.

Despite the extensive and persistent presence of the HIV/AIDS pandemic, and sub-Saharan Africa being the worst affected region globally, it is of concern that a large proportion of our study sample perceived that older persons are at lower risk of contracting the virus than persons in younger age groups; given, moreover, that our study was conducted four decades after the start of the pandemic. While it may be conceded that older persons may indeed be at lower risk of new infection, they remain at risk, nonetheless. Moreover, while their perceived risk is low, their engagement with sexual health information and agencies may neither be high. The fact is that prevention interventions and public health interventions have largely overlooked this population segment. Hence, a lack of tailored information available and appropriately targeted at older persons may serve to confirm, at least for them, that they are not at risk of infection.

This perception of lower risk vulnerability in old age has been recorded in studies conducted more than two decades ago. In 1995 Rose assessed knowledge of AIDS and beliefs about AIDS among 458 senior centre participants aged 60 years and older. Even though the participants acknowledged the gravity of the disease, they tended not to perceive they were susceptible to contracting HIV [55]. Likewise, LeBlanc reported that respondents in his sample contended they did not fear contracting HIV; they perceived they were less likely to be infected than younger individuals, and their chance of being infected was low [56]. Similarly, in a 2019 study in Ghana, Anokye et al. found older participants had only average knowledge and perceptions about HIV/AIDS, despite some living with HIV [12]. The studies suggest, if not indicate, that globally, the older population has been neglected in efforts to address individuals’ vulnerability to HIV infection.

Findings on a relationship between HIV knowledge and gender has not been consistent. Anokye et al. found no association between gender and HIV knowledge [12]. Negin reported lower knowledge in females as compared to males [7]. Henderson et al. reported poor HIV knowledge in 65 per cent of his sample, who were all female [57]. In our study, women had a higher knowledge score than men, probably because older women in the study communities are more likely than older men to attend a senior centre and participate in its activities [58].

As in other studies, education and age similarly influenced the level of knowledge of HIV/AIDS in our study sample. Education was positively associated with knowledge, while age had a negative association [55, 56, 59–61]. In 1991 McCaig and colleagues found that persons aged 50 years and over who did not complete high school had a lower level of knowledge of HIV/AIDS [61]. In 1993 LeBlanc found that when age and knowledge of AIDS were used in path analysis, age remained a significant predictor of knowledge on AIDS, suggesting that older persons had a lower level of knowledge than their younger counterparts [56]. As was the case in our study, Rose conducted an educational intervention study on 458 older adults at senior centres to assess knowledge and perceptions regarding HIV/AIDS [9]. The author provided educational sessions, some of which were delivered by older adults living with HIV. Educational pamphlets were handed out and participants were encouraged to share them with their networks. The participants were fairly knowledgeable about AIDS before the educational intervention, but as in our study, their total knowledge score improved after the programme. Significantly, again as in our study, the perceptions of the participants in Rose’s study regarding their susceptibility to contracting HIV/AIDS and the gravity of HIV/AIDS improved. Rose concluded that age-specific community AIDS educational programmes contextualised facts about HIV in older adults [9], which is an efficient means of providing information to groups of older people, particularly when it is suggested that the information be shared with younger people in their midst. Similarly, as our study participants stated, present methods of imparting education were not impacting behaviour regarding HIV. Consequently, older adults do not perceive themselves as being at risk of contracting HIV.

Health literacy and level of education were low in the majority of our study sample. Poor health literacy is a barrier to seeking and obtaining adequate health care, as such individuals are marginalised in health care systems. In addition, embarrassed by their health illiteracy, these individuals may not volunteer a lack of understanding regarding medications and terminology, increasing a risk of non-compliance, nor ask appropriate questions regarding their health or health condition [62]. A combination of a poorly functioning healthcare system, particularly in rural areas, and low health literacy of persons in the study population, as well as the current focus of HIV/AIDS educational programmes on the younger population are deemed to have contributed to the poor KAP scores we established.

Low health literacy has been shown to correlate with poor HIV/AIDs related knowledge, underscoring a need to tailor HIV prevention strategies toward a population with a high prevalence of inadequate health literacy, in order to refute and dispel misconceptions about HIV/AIDS [63]. Our study indicated strongly that if appropriate education methods are used, knowledge can be improved, hence increasing awareness of risk, prevention and management. In a multi-site study (four sites in the United States, one site in Puerto Rico, and one site in Botswana), Dawson-Rose et al. (2016) found that building long-term relationships and patient-provider trust were central to maintaining adherence and good health in people living with HIV/AIDS, particularly where health literacy is low [64].

Poor health literacy impacts knowledge, attitudes and practices in the case of several health conditions: an example being the current coronavirus (COVID-19) pandemic. Messaging and education on such health pandemics need to be inclusive of an entire population in order to reduce risk of exposure to infection. Varying methods are required to disseminate educational information to sections of a population with differing literacy and health literacy levels.

There are some limitations to the study. While the findings represent a sample of over a thousand who demographically represent much of the older population in South Africa and Lesotho, the study was conducted in a few selected sites and participation was voluntary. The findings may therefore not be generalisable to the older population of the participating countries. The exclusion of the Lesotho sample and the limited access to the Cape Winelands site due to an outbreak of bird flu reduced the sample size of the education intervention. The follow-up intervention had a 69 per cent participation which may have caused bias on the post-intervention results, particularly those of the Cape Winelands. Nonetheless, apart from older age and widowhood, the drop-outs did not differ notably from those who participated in the follow-up study.

## Conclusions

A low level of health literacy was found in the study population. The level of knowledge, attitudes and practices regarding HIV/AIDs was poor, and are a barrier to managing their own risk, and that of their family and community members of contracting the virus. The majority of the participants perceived older people not to be at risk of infection from HIV, which is of concern. Education programmes on HIV/AIDs have largely been targeted at young adults, which adds to older persons’ perception of their not being at risk.

A focused, tailored educational programme targeted at older persons which is easily accessible to them can place them centrally in the fight against the epidemic, even in the presence of low health literacy. The majority of older persons in the two countries head their typically multi-generational household and play a key role in their community; they are both contributors and influencers. Specially tailored educational programmes targeted at older persons will contribute to enhancing their knowledge and modifying their attitudes and behaviour. In turn, the programmes will equip them to provide guidance and support in respect of the disease to younger persons. Educating an older population on HIV/AIDS will benefit both that population segment and the younger population. Hence, policy and educational programmes tailored to meet the information needs of older persons are indicated, that are cognisant of the low health literacy level of a large section of the older population in sub-Saharan Africa.

## Data Availability

Datasets used and analysed for this study are available from the corresponding author on reasonable request.

## Abbreviations

AIDS: Acquired Immunodeficiency Syndrome
AOR: Adjusted odds ratios
COVID-19: Coronavirus disease 2019
CI: Confidence intervals
HIV: Human Immunodeficiency Virus
HL: Health literacy
IAQ-E: International Aids Questionnaire-English Version
KAP: Knowledge, attitudes and practices
OR: Odds ratios
PLWHIV: People living with HIV
SSA: Sub-Saharan Africa
STOFHLA: Short Test of Functional Health Literacy in Adults
UNAIDS: The Joint United Nations Programme on HIV/AIDS
WHO: World Health Organisation
